# A case of intestinal malrotation apparent after laparoscopically total proctocolectomy followed by ileal‐pouch‐anal anastomosis for ulcerative colitis

**DOI:** 10.1111/ases.13114

**Published:** 2022-08-11

**Authors:** Hidetaka Ichikawa, Shinobu Ohnuma, Hirofumi Imoto, Sakiko Kageyama, Minoru Kobayashi, Taiki Kajiwara, Hideaki Karasawa, Atsushi Kohyama, Kazuhiro Watanabe, Naoki Tanaka, Takashi Kamei, Michiaki Unno

**Affiliations:** ^1^ Department of Surgery Tohoku University Graduate School of Medicine Sendai Japan; ^2^ Department of Diagnostic Radiology Tohoku University Graduate School of Medicine Sendai Japan

**Keywords:** intestinal malrotation, total proctocolectomy, ulcerative colitis

## Abstract

Intestinal malrotation (IM) is an abnormality due to a failure of the normal midgut rotation and fixation. We report a case of 46‐year‐old man with ulcerative colitis whose IM was apparent after laparoscopically total proctocolectomy (TPC) followed by ileal‐pouch‐anal anastomosis (IPAA) and ileostomy. There was no abnormal anatomy except for mobile cecum/ascending colon during the initial operation. Intestinal obstruction occurred after ileostomy closure. The computed tomography scan showed the duodeno‐jejunal transition was located in right abdomen, the superior mesenteric vein was located left of the superior mesenteric artery (SMA) and the obstruction point was the distal ileum near the pouch. We performed an ileo‐ileo bypass across the ventral side of the SMA to relieve the intestinal obstruction. The patient would have incomplete IM preoperatively, which became apparent by TPC. In case of TPC for mobile colon, anatomy of small intestine should be checked before IPAA.

## 1 INTRODUCTION

Intestinal malrotation (IM) is a congenital abnormality occurring through failure of normal rotation of the midgut around the superior mesenteric artery (SMA) and fixation in the peritoneal cavity.^1^ Although there have been cases reported of acute abdomen or malignant abdominal disease of IM diagnosed pre‐ or intraoperatively,^2^, ^3^ none have been postoperatively reported. We present a case of postoperative intestinal obstruction caused by IM that was not observed pre‐ or intraoperatively, becoming apparent after laparoscopic total proctocolectomy (TPC) and ileal pouch‐anal anastomosis (IPAA) for ulcerative colitis (UC).

Informed consent was obtained from the patient for the publication of this case report, including medical data and images. Patient anonymity was guaranteed.

## 2 CASE PRESENTATION

A 46‐year‐old man developed UC in his 20s and experienced persistent left‐sided colitis despite medical treatment. He was steroid‐dependent with frequent relapses despite other medical treatments; therefore, he was referred to our department for surgery.

**FIGURE 1:**
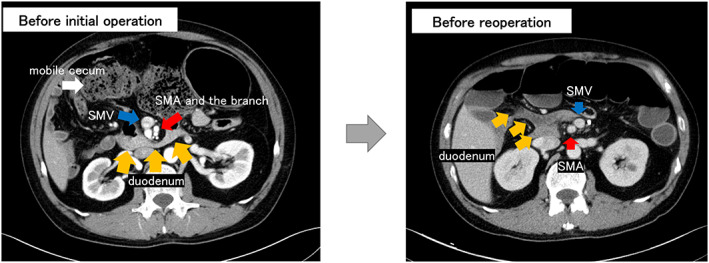
Images of computed tomography scan before initial operation and before reoperation. Before initial operation: the patient had a mobile cecum, and the third portion of the duodenum ran dorsal to the superior mesenteric artery (SMA) and superior mesenteric vein (SMV). Before reoperation: the duodeno‐jejunal transition was located on the right side of the abdomen, and the SMV was located to the left of the SMA

Preoperative computed tomography (CT) scan revealed that the cecum and ascending colon were unattached to the lateral peritoneum; however, the third portion of the duodenum normally ran dorsal to the SMA and superior mesenteric vein (SMV) (Figure 1). There were no anatomical abnormalities in the SMA and SMV morphology. The transverse to descending colon was dilated and accompanied by inflammation.

**FIGURE 2:**
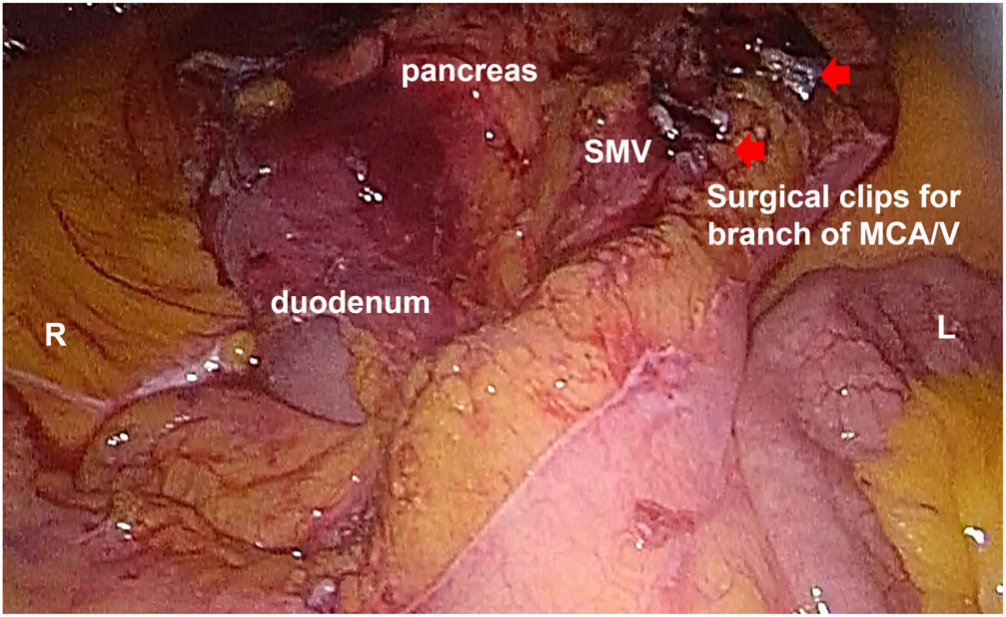
Laparoscopic upper‐abdominal view at the initial operation. The superior mesenteric vein (SMV) was located to the right of the superior mesenteric artery (SMA), and the third portion of the duodenum was located dorsal to them. L, left side of the patient; MCA/V, middle colic artery/vein; R, right side of the patient. Arrows show surgical clips for MCA/V

We performed laparoscopic TPC followed by J‐shaped IPAA and construction of a diverting loop ileostomy. The surgery was preceded by rectal mucosal removal, followed by laparoscopic procedures. Adhesions were not observed in the abdominal cavity. We initiated the laparoscopic procedure of the rectosigmoid colon and resected the inferior mesenteric artery. The descending colon was dissected from the retroperitoneum. We opened the omental bursa cavity exposing the inferior border of the pancreas, isolated the mesentery of the transverse colon and detached the splenic flexure colon. The inferior mesenteric vein was resected from the inferior border of the pancreas. The right and left branches of the middle colic artery, middle colic vein, accessory right colic vein, and right colic artery were resected. As the cecum and ascending colon were not fixed to the retroperitoneum, there was no need to detach them. We enlarged the umbilical port wound, led the entire colon out of the abdominal cavity, preserved the ileocolonic artery/vein, and cut the end ileum using a linear stapler to create a J‐shaped ileal pouch. After confirmation that there was no abnormality in the anatomical placement of the small intestines (Figure 2), the pouch was guided to the anus without torsion of the small bowel mesentery during the laparoscopic procedure. We constructed a hand‐sewn regular IPAA and a loop ileostomy 50 cm from the anastomotic site.

The patient was discharged on the 15th postoperative day without complications. The ileostomy was closed after 2 months. Intestinal obstruction occurred afterwards. During the diagnostic workup, the CT scan showed that the duodeno‐jejunal transition was in the right abdomen, the SMV was to the left of the SMA, known as the SMV rotation sign^4^ (Figure 1). The CT scan also showed that the duodenal third portion was not running dorsal to the SMA and SMV (Figure S1), and the obstruction point was the distal ileum near the pouch. We suspected that the patient had IM, which was not apparent at the time of surgery, and that torsion of the ileum based on IM caused intestinal obstruction.

**FIGURE 3:**
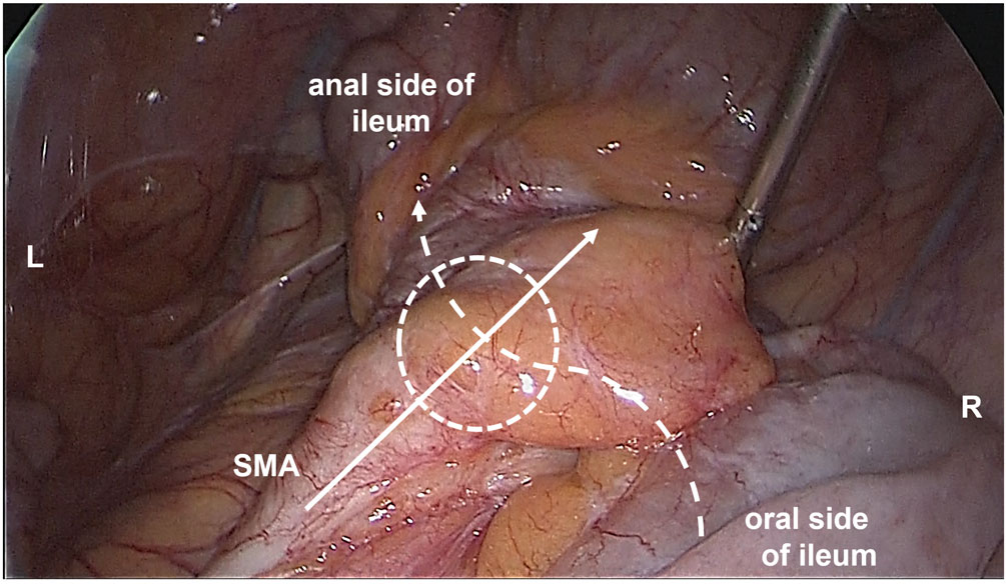
Laparoscopic pelvic view at the reoperation. Most of the small intestine passed through the dorsal side of the SMA from the left side to the right side of the patient, and that the distal ileum was compressed by the SMA near the pouch. L, left side of the patient; R, right side of the patient; SMA, superior mesenteric artery. White line with arrow shows the running of the SMA. Dotted line with arrow shows the running the ileum. Dotted circle shows the point of intestinal obstruction

**FIGURE 4:**
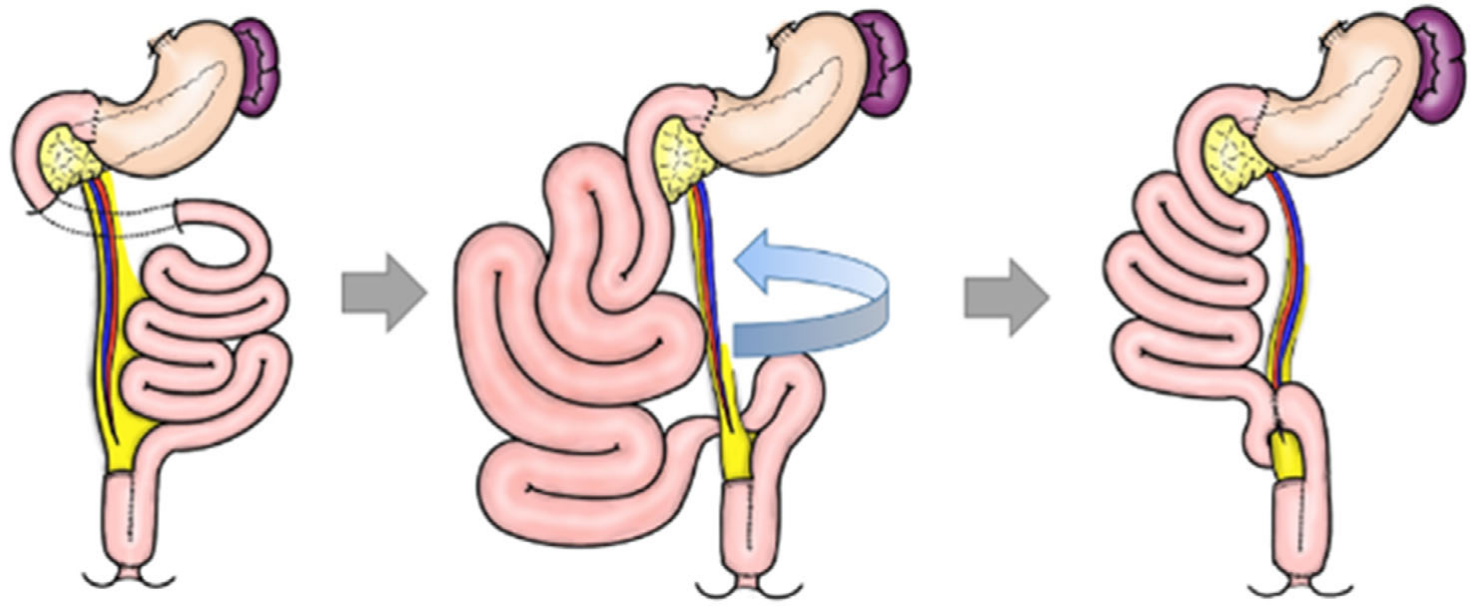
Schema of the cause of intestinal obstruction and reoperation. Left: state after total proctocolectomy and ileal‐pouch anal anastomosis in patients without intestinal malrotation. Center: because the duodenum was not fixed to the retroperitoneum, most of the small intestine passed through the dorsal superior mesenteric artery (SMA) from the left side to the right side, and the distal ileum was compressed by the SMA near the pouch. Right: an ileo‐ileo bypass was performed across the ventral side of the SMA

We performed laparoscopic reoperation and found that most of the small intestine passed through the dorsal side of the SMA from the left side to the right side, and that the distal ileum was compressed by the SMA near the pouch. Furthermore, we confirmed that the duodenum was not fixed to the retroperitoneum and was running ventral to the SMA and SMV. By converting to laparotomy, we performed ileo‐ileo bypass across the ventral side of the SMA (Figures 3 and 4), and the patient's symptoms improved. He has been free of intestinal obstruction for 4 years.

## 3 DISCUSSION

During the embryonic period, the duodenal jejunal loop passes under the SMA, the lower ileum and cecum/ascending colon pass over the SMA and are in the right upper quadrant, forming the ligament of Treitz. The cecum descends into the right lower abdomen, and the cecum and ascending colon affix to the retroperitoneum. The mesenteric root of the small intestine affixes obliquely from the ligament to the right, inferiorly toward the ileocecal region. IM is a congenital abnormality occurring because of inadequacy of this process.^1^ Typical signs are right‐sided small bowel, left‐sided colon, and abnormal relationship of the SMV to the left of the SMA instead of to the right, known as the SMV rotation sign.^4^ Historically, IM has been considered a childhood disease, although several adult cases have been reported in patients with colorectal cancer and laparoscopic surgery.^2^, ^3^

UC is a diffuse, nonspecific inflammation of unknown origin that continuously damages the colonic mucosa from the rectal side oftentimes leading to erosions and ulcers. The absolute indications for surgery for UC include colonic perforation, massive bleeding, toxic megacolon, high‐grade dysplasia, and cancer. Steroid‐dependent cases, cases refractory to other medical treatments, and cases of low‐grade dysplasia detected by biopsy are relative indications for surgery.^5^, ^6^ The combination of TPC and IPAA is the gold standard technique for UC since TPC is highly curative due to the non‐remnant colonic mucosa, and IPAA can preserve anal function.^7^, ^8^ Additionally, laparoscopic surgery for UC can be considered a safe and reliable technique for short‐and long‐term outcomes.^9^ However, one of the complications after TPC + IPAA is the intestinal obstruction, which is caused by adhesion, hernia, and postoperative rotation of the small intestine, and so forth.

As far as we know, this is the first case report of postoperative manifestations of IM which caused an intestinal obstruction. We presume that this patient had a malformation of the Treitz ligament; however, his small intestine was running like a normal anatomy, (i.e., the third portion of the duodenum passed dorsal to the SMA), and that by detaching the mesentery root of the transverse colon from the inferior border of the pancreas, the IM became apparent after TPC + IPAA. As a result, most of the small intestine rotated clockwise around the SMA (i.e., the reverse of fetal rotation), and the ileum near the IPAA was compressed by the SMA, resulting in intestinal obstruction. Although the small intestinal mesentery without torsion was carefully checked, IM was not diagnosed during initial surgery. We performed an ileo‐ileo bypass with side‐to‐side anastomosis across the SMA to clear the intestinal obstruction. If IM becomes apparent during initial surgery, it is better to position the ileum while keeping it on the right side of the SMA, as in Ladd's operation.^10^ The IPAA should then be performed in the style of an inverted J‐pouch.

In conclusion, in cases of TPC for a mobile right‐sided colon, it is necessary to confirm the strength of the ligament of Treitz and mesentery root of the small intestine before IPAA. If IM is apparent, IPAA with an inverted J‐pouch would be a solution for preventing torsion.

## CONFLICT OF INTEREST

The authors declare they have no conflicts of interest.

## Supporting information


**FIGURE S1** 3D computed tomography scan before the reoperation The duodeno‐jejunal transition was located on the right side of the abdomen, and the SMV was located to the left of the SMA. The duodenal third portion is not running dorsal to the SMA and SMV. MCA/V, middle colic artery/vein; SMA/V, superior mesenteric artery/vein

## Data Availability

The data that supports the findings of this study are available in the supplementary material of this article
