# Supplementary figures and images for: A case of intestinal malrotation apparent after laparoscopically total proctocolectomy followed by ileal‐pouch‐anal anastomosis for ulcerative colitis
Source: Asian J Endosc Surg. 2022 Aug 11;16(1):114–7. doi: 10.1111/ases.13114 (PMC10087278; doi:10.1111/ases.13114)

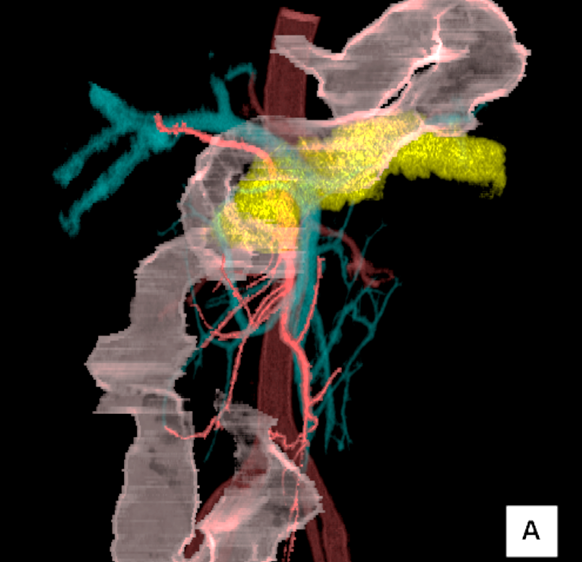

Supplement: Supplementary file 1 — FIGURE S1 3D computed tomography scan before the reoperation The duodeno‐jejunal transition was located on the right side of the abdomen, and the SMV was located to the left of the SMA. The duodenal third portion is not running dorsal to the SMA and SMV. MCA/V, middle colic artery/vein; SMA/V, superior mesenteric artery/vein [file ASES-16-114-s001.tif]
